# AMPKα1 confers survival advantage of colorectal cancer cells under metabolic stress by promoting redox balance through the regulation of glutathione reductase phosphorylation

**DOI:** 10.1038/s41388-019-1004-2

**Published:** 2019-09-17

**Authors:** Ying-Nan Wang, Yun-Xin Lu, Jie Liu, Ying Jin, Hui-Chang Bi, Qi Zhao, Ze-Xian Liu, Ying-Qin Li, Jia-Jia Hu, Hui Sheng, Yi-Ming Jiang, Chao Zhang, Feng Tian, Yang Chen, Zhi-Zhong Pan, Gong Chen, Zhao-Lei Zeng, Kai-Yan Liu, Marcia Ogasawara, Jin-Ping Yun, Huai-Qiang Ju, Jian-Xiong Feng, Dan Xie, Song Gao, Wei-Hua Jia, Scott Kopetz, Rui-Hua Xu, Feng Wang

**Affiliations:** 1 Sun Yat-sen University Cancer Center; State Key Laboratory of Oncology in South China, Collaborative Innovation Center for Cancer Medicine, Guangzhou, 510060 Guangdong China; 2 Department of Biomedical Engineering, School of Engineering, Sun Yat-sen University, Guangzhou, 510006 Guangdong China; 3 Department of Medical Oncology, Sun Yat-sen University Cancer Center, 510060 Guangzhou, China; 4 School of Pharmaceutical Sciences, Sun Yat-sen University, Guangzhou, 510006 Guangdong China; 5 Department of Pathology, Sun Yat-sen University Cancer Center; State Key Laboratory of Oncology in South China, Collaborative Innovation Center for Cancer Medicine, Guangzhou, 510060 Guangdong China; 6 Department of Gastrointestinal Medical Oncology, University of Texas MD Anderson Cancer Center, Houston, TX 77030 USA; 7 Department of Colorectal Surgery, Sun Yat-sen University Cancer Center; State Key Laboratory of Oncology in South China, Collaborative Innovation Center for Cancer Medicine, Guangzhou, 510060 Guangdong China; 8 Department of Experimental Therapeutics, University of Texas MD Anderson Cancer Center, Houston, 77030 TX USA

**Keywords:** Cancer metabolism, Apoptosis

## Abstract

Patients with stage II or III colorectal cancer (CRC) exhibit various clinical outcomes after radical treatments. The 5-year survival rate was between 50 and 87%. However, the underlying mechanisms of the variation remain unclear. Here we show that AMPKα1 is overexpressed in CRC patient specimens and the high expression is correlated with poor patient survival. We further reveal a previously unrecognized function of AMPKα1, which maintains high level of reduced glutathione to keep reduction–oxidation reaction (redox) homeostasis under stress conditions, thus promoting CRC cell survival under metabolic stress in vitro and enhancing tumorigenesis in vivo. Mechanistically, AMPKα1 regulate the glutathione reductase (GSR) phosphorylation possibly through residue Thr507 which enhances its activity. Suppression of AMPKα1 by using nano-sized polymeric vector induces a favorable therapeutic effect, especially when in combination with oxaliplatin. Our study uncovers a novel function of AMPKα1 in redox regulation and identifies a promising therapeutic strategy for treatment of CRC.

## Introduction

Colorectal cancer (CRC) is the third most prevalent cancer among males and the second most prevalent among females worldwide [1]. The incidence and mortality rates of CRC in China have been progressively increasing due to changes in lifestyle and diet [2–5]. Survival rates for CRC can vary based on a variety of factors, particularly clinical stage. The 5-year survival rate of patients with localized cancer (stage I) is ~95%. However, if the cancer has spread to distant parts of the body (stage IV), the 5-year survival rate is 13%. For patients with stage II/III CRC, the prognosis varies even under similar treatments, with a 5-year survival rate between 50 and 87% [6]. Nevertheless, both the American Joint Committee on Cancer (AJCC) sixth edition and seventh edition staging systems do not address all survival discrepancies in CRC [7–9]. Therefore, determining other prognostic factors and underlying mechanisms for differences in survival are critical for making decisions about therapy.

AMP-activated protein kinase (AMPK) is a heterotrimeric serine/threonine protein kinase, consisting of α (catalytic), β and γ (regulatory) subunits, where phosphorylation of T172 in the α-catalytic subunit is a critical event for its full activation [10]. AMPK has a central role in the regulation of cellular metabolism and energy homeostasis in mammalian tissues [11]. Once activated, AMPK maintains energy balance through the activation of catabolism to promote ATP production and the inhibition of anabolism to conserve ATP [12]. The role of AMPK in cancer has been hotly debated. AMPK had previously been regarded as a tumor suppressor, as evidenced by the demonstration that it negatively regulates aerobic glycolysis in cancer cells and suppresses tumor growth in vivo [13–15]. AMPK activators such as metformin have been shown to suppress tumor cell proliferation both in vitro and in vivo [16, 17]. Deletion of the α1 catalytic subunit of AMPK accelerates the development of lymphoma in transgenic mice overexpressing *C-MYC* in B cells [13]. In contrast, AMPK is critical for promoting cancer cell survival under energy stress by maintaining NADPH levels [18]. AMPK also confers metabolic stress resistance to leukemia-initiating cells and promotes leukemogenesis [19]. Previous studies were mostly performed in cell lines and mouse models, it is important to identify the function of AMPK from a clinical standpoint and to clarify its underlying mechanism.

Here, we demonstrate that high levels of AMPKα1 are correlated with a poor prognosis in CRC patients and inhibition of AMPKα1 profoundly kills CRC cells by attenuating glutathione metabolism. In addition, we revealed that AMPKα1 regulates glutathione reductase phosphorylation possibly through residue Thr507 which enhances its activity. Downregulating the expression of AMPKα1 by using optimized poly(amine-*co*-esters)/short hairpin RNA (shRNA) polyplexes is able to significantly inhibit tumor growth in CRC mice models. Our results reveal the function of AMPKα1 and indicate that AMPKα1 can be a novel target in CRC.

## Results

### AMPKα1 is significantly correlated with poor patient survival

**Fig. 1:**
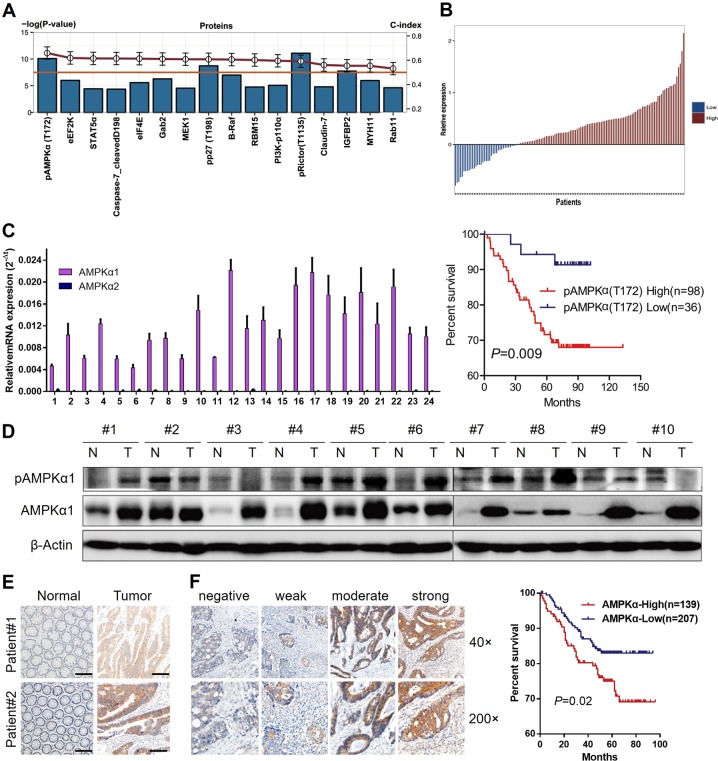
Identification of AMPKα1 as prognostic predictor for stage II/III CRC patients. **a** 16 proteins were significantly correlated with overall CRC survival (univariate Cox regression analysis, Wald *P* value < 0.05, shown in bar plot). C-index values are also summarized in the line plot (red). pAMPK(T172) had the highest C-index (0.659). Proteins from fresh cancer and normal tissues were detected using the RPPA platform. **b** Patients were assigned into high expression group and low expression group according to pAMPK(T172) expression (Upper panel). Kaplan–Meier (KM) survival curves of 134 CRC patients are stratified by expression levels of pAMPK(T172) (Log-rank test, *P* = 0.009) (Lower panel). **c** mRNA expression of AMPKα1 was examined with qRT-PCR and indicated an upregulation in cancer tissues of 24 CRC patients compared with their nontumorous tissues. **d** Immunoblotting of AMPKα1 and pAMPK(T172) in ten paired nontumorous tissues and cancer tissues. **e** Immunohistochemistry of AMPKα1 in two representative paired nontumorous tissues and cancer tissues. Scale bar: 100 μm. **f** Left: Immunohistochemistry of AMPKα1 in patients with different stages of CRC cancer. Representative images are shown. Right: Kaplan–Meier (KM) survival curves of 346 CRC patients are stratified by AMPKα1 immunohistochemistry scores

Using a reverse phase protein array (RPPA) assay, we quantified 141 cancer-relevant proteins and phosphoproteins in 134 paired samples from patients with stage II/III CRC. The clinical characteristics of those patients are summarized in Table S1. Univariate Cox regression analysis showed that 16 proteins were significantly correlated with overall survival (Table S2). Among those proteins, pRictor (T1135) and pAMPK (T172) were highlighted (*P* < 0.001). Meanwhile, the concordance indexes (C-indexes) of the variables were summarized to investigate the discriminatory ability of random effects for overall survival status. Interestingly, the C-index of pAMPK (T172) (0.659) is much higher than that of pRictor (T1135) (0.591) and the other proteins (0.595–0.619) analyzed, which suggests AMPK as a prognostic predictor in our study group (Fig. 1a). Using the difference in expression of pAMPK (T172) between tumors and paired normal tissues as the cutoff (Fig. 1b), we also found that patients with higher relative expression of pAMPK (T172) in tumor tissues showed much lower overall survival rates than those with lower expression of pAMPK (T172) in tumor tissues (log-rank test, *P* = 0.009, Fig. 1b). Furthermore, the hazard ratio of pAMPK (T172) was 2.376 (95% CI: 1.359–4.152, *P* = 0.0002), indicating that pAMPK (T172) is likely to play an independent role in determining the risks in stage II or III CRC (Table S3).

The RPPA analysis results suggest that AMPKα protein might be correlated with overall survival of stage II/III CRC patients. Since phosphorylation of Thr172 in the AMPKα subunit is a critical event for its full activation, we thus assessed protein levels of AMPKα1, AMPKα2, and pAMPK (T172) and mRNA levels of AMPKα1 in different CRC cell lines. As expected, both the protein and mRNA levels of AMPKα1 were higher in almost all tumor cells compared with the levels in normal colon cell lines CCD112, CCD841, and NCM460 (Fig. S1A, B). In addition, we have also tested the mRNA expression of AMPKα1 and AMPKα2 in 24 CRC patients’ tumor samples, in which AMPKα1 showed much higher expression than AMPKα2 in all tumor samples (Fig. 1c). In addition, protein levels of AMPKα1 and pAMPK (T172) were higher in most cancer samples compared with that in paired nontumorous tissues (Fig. 1d), which was further validated with immunohistochemistry assays (Fig. 1e).

Therefore, we further explored the prognostic performance of AMPKα1 in a larger cohort of CRC patients that contained not only stage II/III but also stage I/IV. These 346 patients contained the previous 140 patients and comprised of 23 stage I, 144 stage II, 128 stage III, and 51 stage IV patients. Consistent with the RPPA findings, patients with higher expression levels of AMPKα1 showed much lower overall survival rates than those with lower expression levels of AMPKα1 (Log-rank test, *P* = 0.02, Fig. 1f). We concluded that the high levels of AMPKα1 were significantly correlated with poor overall survival in stage II/III CRC.

### AMPKα1 is required for CRC cell survival and tumor growth

**Fig. 2:**
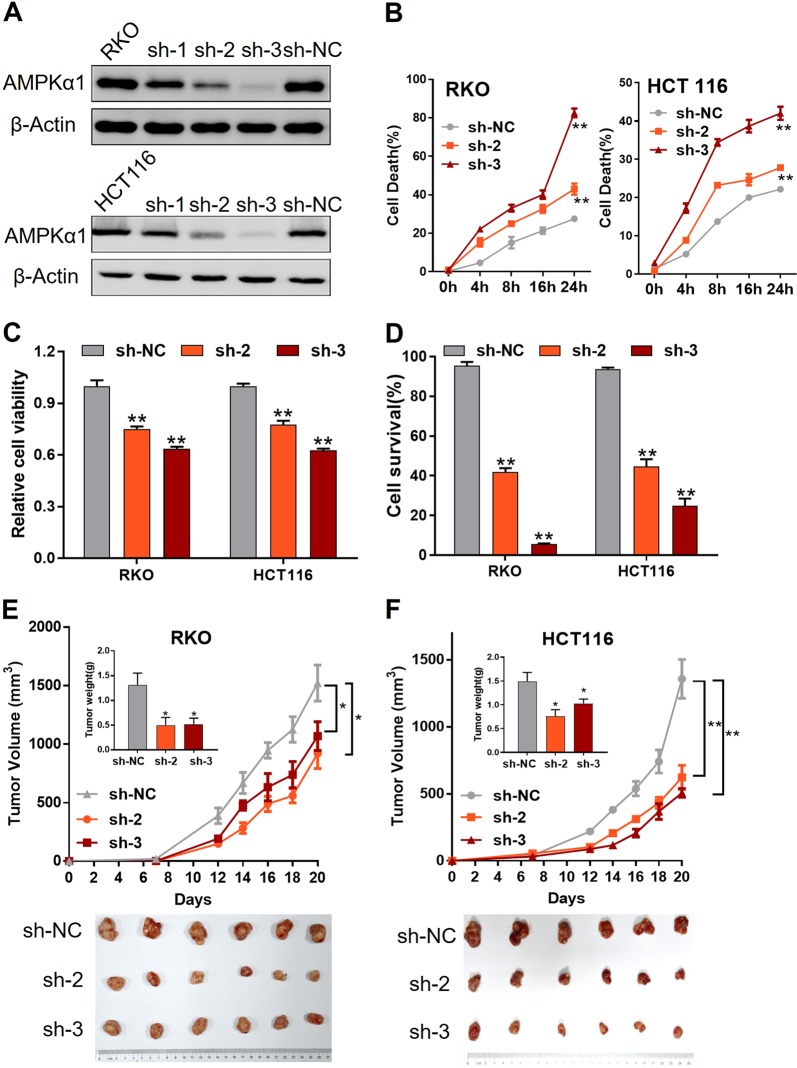
Expression of AMPKα1 maintains tumor cell survival in the absence of glucose. **a** Immunoblotting of AMPKα1 transfected with lentiviruses expressing different shRNAs in RKO and HCT116 showed that sh-3 had the highest efficiency and sh-2 the median. **b** RKO and HCT116 cells expressing control-shRNA (sh-NC), sh-2 RNA, or sh-3 RNA are cultured in glucose-free medium for the indicated time points to quantify cell death via a trypan exclusion assay. **c** Viability of RKO and HCT116 cells was detected by CCK-8 assay in the absence of glucose for 24 h. **d** Apoptosis of RKO and HCT116 cells cultured in the absence of glucose for 24 h was detected by flow cytometry. RKO (**e**) and HCT116 (**f**) cells expressing control-shRNA (sh-NC), sh-2 RNA, or sh-3 RNA were subcutaneously injected into the right armpit of nude mice and tumor volumes were recorded three times per week. Tumor tissues dissected from each group were photographed. *P < 0.05, **P < 0.01, Student’s *t*-test

To examine the function of AMPKα1 in CRC cells, we generated stable AMPKα1 knockdown RKO and HCT116 cells by using shRNA (Fig. 2a) and then challenged cells with glucose-free medium. Compared with their parental counterparts, cells expressing shRNA targeting AMPKα1 underwent greater cell death and impaired cell viability in RKO and HCT116 cells (Fig. 2b, c). In addition, similar results were observed in other two CRC cell lines SW1116 and DLD1 by using siRNA (Fig. S2). Consistent with these results, more apoptotic cells were detected in the knockdown group than in their counterparts (Fig. 2d). To determine tumorigenic effect of AMPKα1 in vivo, we injected the RKO and HCT116 cells with control or AMPKα1-shRNAs subcutaneously into nude mice and found that AMPKα1 knockdown significantly inhibited tumor growth (Fig. 2e, f). Taken together, these results demonstrate that AMPKα1 is required for CRC cell survival and tumor growth under conditions of energy stress in vitro and in vivo.

### AMPKα1 maintains cellular glutathione levels

**Fig. 3:**
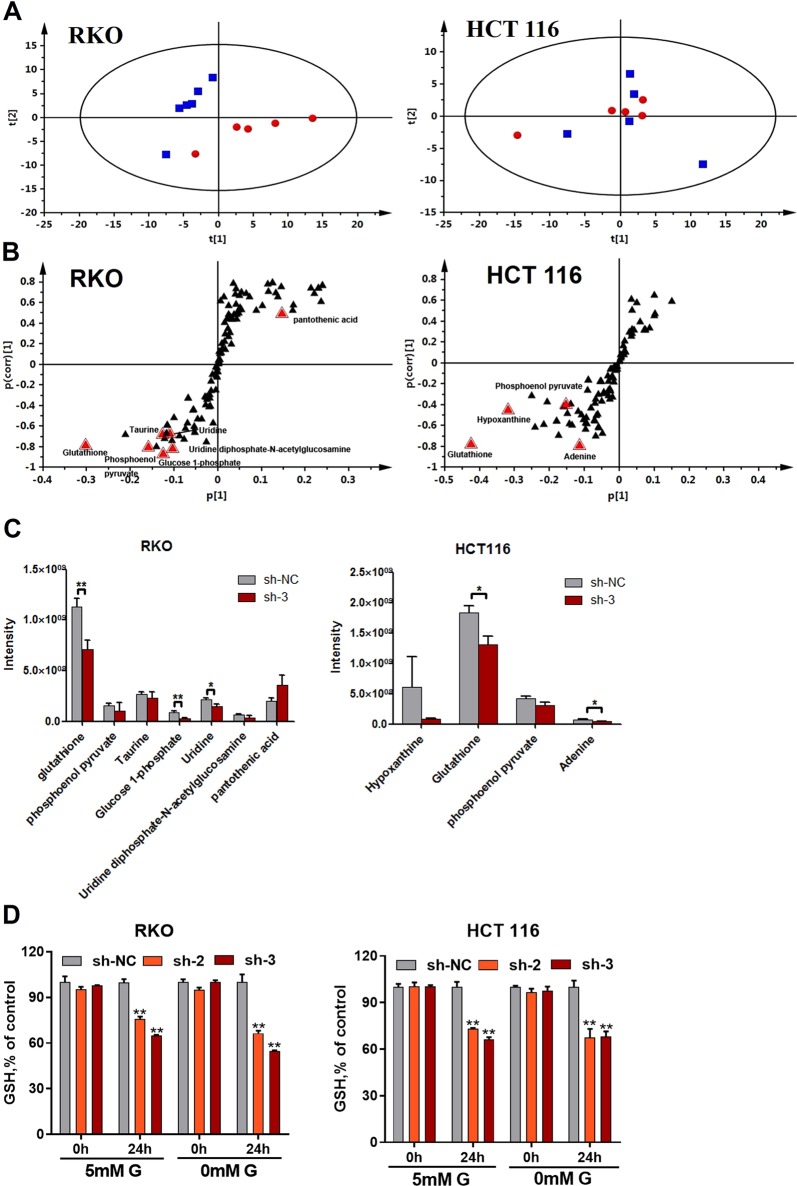
Expression of AMPKα1 maintains cellular glutathione levels. **a** Scatter plot of scores from the PCA analysis of RKO and HCT116 cells expressing control-shRNA (sh-NC, blue boxes) or #3-shRNA (sh-3, red dots) under the negative ionization mode. **b** S-plot of OPLS-DA analysis of RKO and HCT116 cells expressing control-shRNA (sh-NC) or #3-shRNA (sh-3). **c** Relative cellular abundance of metabolites was different in RKO and HCT116 cells expressing control-shRNA (sh-NC) or #3-shRNA (sh-3). **d** Identification of cellular GSH depletion in RKO and HCT116 cells expressing control-shRNA (sh-NC) or #3-shRNA (sh-3) incubated in 5 mM glucose or glucose-free medium. Data are presented as the mean ± SD (*n* = 6). *P < 0.05, **P < 0.01, Student’s *t*-test

To determine how AMPKα1 affects cellular energy metabolism and regulates CRC cell survival, a metabolomic analysis was performed on HCT116 and RKO cell lines. Unsupervised principal components analysis revealed that the principal components of the metabolomic profiles obtained from the HILIC-ESIESI^−^-MS analysis did not show a separation of control NC-shRNA treated HCT116 cells from the AMPKα1-shRNA3-treated HCT116 cells, whereas the principal components in RKO cells did show an improved segregation (Fig. 3a). Supervised orthogonal partial least squares discriminant analysis (OPLS-DA) successfully discriminated between the NC-shRNA and AMPKα1-shRNA3-treated HCT116 or RKO cells (data not shown). Further analysis of the significant discriminatory metabolites was screened using variable importance in the projection scores (VIP > 1) (Fig. 3b). The relative responses of glutathione (*P* < 0.01), glucose 1-phosphate (*P* < 0.01), and uridine (*P* < 0.05) were significantly lower in AMPKα1 knockdown RKO cells (Fig. 3c). Moreover, the levels of glutathione were also lower in AMPKα1 knockdown HCT116 cells (Fig. 3c). Consistent with these results, AMPKα1 knockdown in RKO or HCT116 cells significantly decreased cellular glutathione levels (Fig. 3d).

### AMPKα1 maintains ROS at low levels through the regulation of GSR phosphorylation

**Fig. 4:**
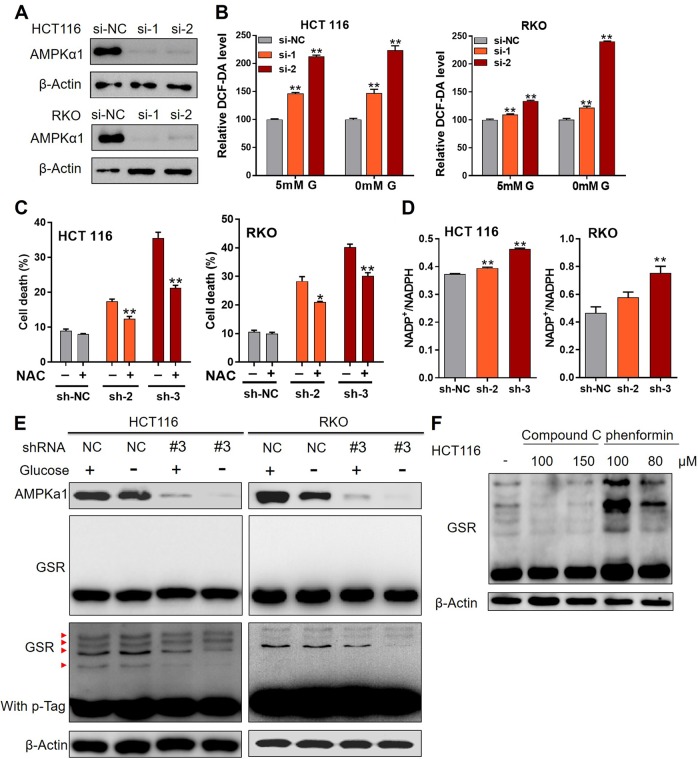
Phosphorylation of GSR by AMPKα1 was responsible for ROS maintenance. **a** Knockdown efficiency of siRNA targeting AMPKα1 was validated by western blot analysis. **b** RKO and HCT116 cells were transfecting with siRNA targeting AMPKα1 before incubation with 5 mM glucose or glucose-free medium and the cellular ROS production was detected with DCF-DA staining. **c** RKO and HCT116 cells expressing control-shRNA (sh-NC), sh-2 RNA, or sh-3 RNA were cultured in glucose-free medium with or without NAC (3 mM) for 18 h before cell death was quantified by trypan exclusion staining. **d** RKO and HCT116 cells expressing sh-NC, sh-2 RNA, or sh-3 RNA were cultured in glucose-free medium for 12 h before measurement of the intracellular NADP^+^/NADPH ratio. **e** Total cell lysates from RKO and HCT116 cells cultured with or without glucose were subjected to Phos-tag gel electrophoresis and immunoblotted for GSR. pGSR: red triangle. Top shows immunoblot for GSR without Phos-tag. **f** Total cell lysates from HCT116 cells treated with Compound C or phenformin were subjected to phos-tag gel electrophoresis and immunoblotted for GSR. *P < 0.05, **P < 0.01, Student’s *t*-test

Glutathione is the most abundant cellular antioxidant, we then assessed cellular reactive oxygen species (ROS) levels. AMPKα1 knockdown with siRNAs (Fig. 4a) dramatically increased total cellular ROS levels after glucose shortage (Fig. 4b). N-acetyl-cysteine (NAC), a precursor for glutathione synthesis, effectively suppressed cell death in AMPKα1 knockdown cells (Fig. 4c). Consistently, higher NADP^+^/NADPH ratios were observed in AMPKα1 knockdown CRC cells compared with the ratios in NC shRNA cells, which reflects NADPH depletion (Fig. 4d). Since AMPKα1 is a kinase and could phosphorylate enzymes, we proposed that glutathione synthesis might be regulated by AMPKα1-dependent phosphorylation. Addition of phosphor-tag into SDS-PAGE gels showed that several residues of glutathione synthesis reductase (GSR) were phosphorylated and the phosphorylation bands were weakened after silence of AMPKα1 (Fig. 4e). Consistently, treatment with Compound C or phenformin significantly suppressed or activated GSR phosphorylation in HCT116 cells (Fig. 4f).

**Fig. 5:**
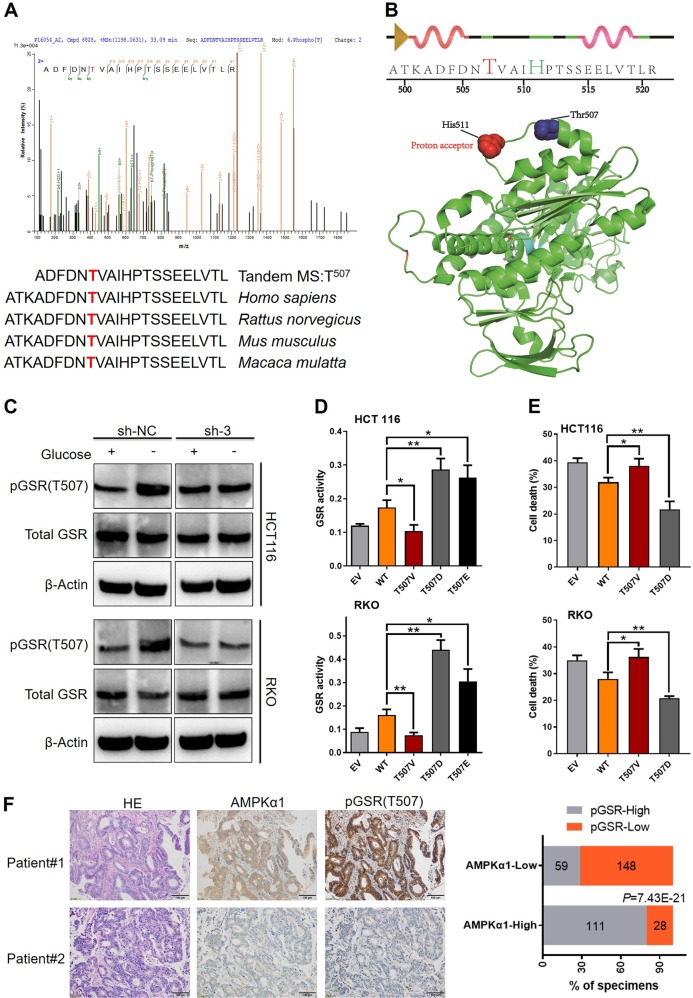
Thr507 phosphorylation of GSR is important for the survival function of AMPKα1. **a** MS/MS spectra of phosphopeptides containing the phosphotyrosine (pThr) 507 site of GSR. Fragment ions are shown, as is the sequence coverage due to identified fragment ions. All of the highest peaks were explained, but for clarity, they are not all annotated. m/z, mass-to-charge ratio (Upper panel). Thr507 phosphorylation site is highly conserved among human, rat, mouse, and monkey. Identified phosphopeptide are shown above the sequence alignments (Lower panel). **b** The identified peptide containing the phosphorylated site was shown in two dimension. The phosphorylated site Thr507 and the proton acceptor His511 were indicated in red and green, respectively (Upper panel). Thr507 in the crystal structure of human GSR. Overall view of GSR (Protein Data Bank ID code 3dk8) was shown. The relative positions of His511 (red spheres) and Thr507 (cyan spheres) are indicated (Lower panel). **c** Immunoblotting of pGSR(T507), total GSR in RKO, and HCT116 cells cultured in glucose deprivation conditions. **d** GSR enzymatic activities of T/V, T/D, and T/E mutations of Thr507 in HCT116 and RKO cells. **e** Cell apoptosis of T/V and T/D upon glucose shortage was determined by annexin V/PI staining. **f** Left: Immunohistochemistry of AMPKα1 and pGSR(T507) in CRC clinical samples. Representative images including corresponding hematoxylin and eosin staining are shown. Right: Scoring of pGSR(T507) in AMPKα1 low and high samples and correlation analysis by using Chi-square test. Results are representative of three experiments. *P < 0.05, **P < 0.01, Student’s *t*-test, Chi-square test

To further confirm the phosphorylated residues of GSR, HPLC-tandem mass spectrometry (MS/MS) analysis was performed on tryptic digests of GSR immunoprecipitants from HCT116 transfected with lentivirus expressing NC or AMPKα1-shRNA after glucose deprivation. MS spectra showed residue Thr507, which is highly conserved among species, was found only in HCT116 NC-shRNA cells, but not found in AMPKα1-shRNA cells (Fig. 5a). According to the structure of GSR (Protein Data Bank ID: 3dk8), Thr507 lies adjacent to His511, proton acceptor of GSR, whereby phosphorylation might play critical roles in its activity (Fig. 5b). We next produced specific antibody recognizing phosphorylation of Thr507 in GSR (pGSR). Figure 5c demonstrated that glucose deprivation induced Thr507 phosphorylation of GSR was AMPKα1-dependent, while total GSR levels were not affected by AMPKα1 expression. To further determine phosphorylation of Thr507 in GSR was involved in glucose stress resistance, we mutated Thr507 into the phosphorylation-imitated Asp507 (T507E) and Glu507 (T507D) and negative control Val507 (T507V) and then transfected them in AMPKα1 and GSR double knockdown HCT116 and RKO cells (Fig. S3). Enzymic activity assays revealed that compared with cells expressing the GSR-WT, GSR activity was decreased in cells transfected with the T507V mutation vector and enhanced in cells transfected with the T507E and T507D mutation vector (Fig. 5d). To test the ability of the GSR Thr507 phosphorylation to support the survival of HCT116 and RKO cells in AMPKα1 knockdown cells upon glucose shortage, we performed flow assays to detect the apoptotic proportion in these cells. The results showed that GSR T507D mutant partly reversed CRC cell death caused by AMPKα1 and GSR double knockdown (Fig. 5e). In the clinical samples, immune-staining with pGSR correlated positively with AMPKα1 expression (Fig. 5f). Collectively, these results indicated that GSR Thr507 is most likely the inducible phosphorylation substrates of AMPKα1 in CRC cells under glucose deprivation and that it plays critical roles in GSR activity and likely functions accounting for the protective roles of AMPKα1 in cell survival under nutrient stress.

### AMPKα1 is a potential target in CRC

**Fig. 6:**
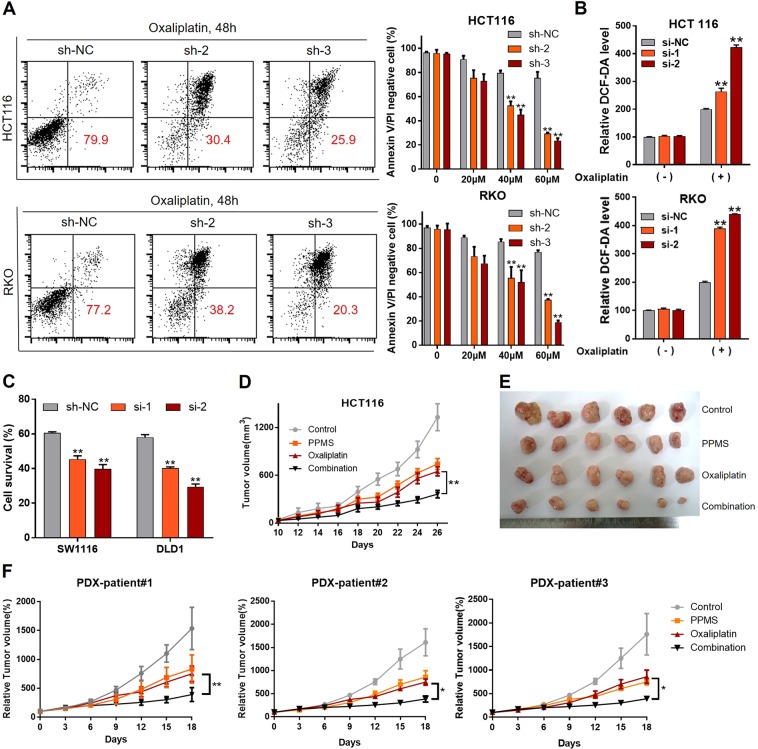
Inhibition of AMPKα1 is synergistic with oxaliplatin. **a** Cell apoptosis in HCT116 and RKO cells expressing control-shRNA (sh-NC), sh-2 RNA, or sh-3 RNA after oxaliplatin treatment. Representative images and quantification are shown. **b** Intracellular ROS levels in HCT116 and RKO cells were detected with DCF-DA. **c** Cell survival of SW1116 and DLD1 cells treated by oxaliplatin (60 μM) was measured by CCK-8 assay. **d** Tumor volumes after tail vein administration of different formulas until day 26. **e** The images of HCT116 tumor tissue after indicated treatments. **f** Tumor volumes after tail vein administration of different formulas until day 18 in a PDX model. *P < 0.05, **P < 0.01, Student’s *t*-test

Chemotherapeutic agents were previously reported to induce redox stress in cancer cells [20]. We therefore hypothesized that knockdown of AMPKα1 could sensitize CRC cells to oxaliplatin treatment. Flow assays showed increased cell apoptosis after silence of AMPKα1 when treated with oxaliplatin in HCT116 and RKO cells (Fig. 6a). Treatment with oxaliplatin induced significant elevated ROS levels in AMPKα1 knockdown cells (Fig. 6b). In addition, we detected the IC50 of oxaliplatin in 10 CRC cell lines (Table S4). We found HCT116 and RKO cell lines are relatively sensitive to oxaliplatin. We also evaluated the effect of AMPKα1 knockdown on oxaliplatin sensitivity in oxaliplatin-resistant SW1116 and DLD1 cells. The results showed that this effect was not cell line dependent (Fig. 6c). In order to confirm the in vitro results, we constructed a shRNA delivery system, HA-quaternary polyplex, to validate the therapeutic effects of AMPKα1 knockdown in vivo. The characteristics and function of the polyplex was confirmed in Fig. S4. The AMPKα1-shRNA3 treatment groups result in significant inhibition of tumor growth compared with the control group (Fig. 6d, e and S5A). Moreover, the body weight and histological analysis of organs of mice treated with in PPMS polyplexes did not differ from that of the control, indicating that the PPMS polyplexes did not exhibit severe systemic toxicity (Fig. S5B, C). In addition, the specific downregulations of AMPKα1 on both mRNA and protein levels were observed in the PPMS polyplexes/AMPKα1-shRNA3 treated mice group (Fig. S5D, E). Besides PPMS polyplexes, AMPK inhibitor Compound C and oxaliplatin were used for combination therapy in mouse model. The results showed that Compound C could enhance the sensitivity of CRC tumors to oxaliplatin treatment (Fig. S6).

To further explore the relationship between AMPKα1 expression and oxaliplatin efficacy in clinical data, IHC staining for AMPKα1 was performed on tumor tissues from 76 patients with advanced CRC treated with FOLFOX or XELOX regimens as first line therapy. Overall 27 of 32 (84.3%) patients with low AMPKα1 expression in their primary tumors benefited (CR + PR + SD) from chemotherapy, whereas only 18 of 44 (40.9%) patients with high AMPKα1 expression benefited from the therapy (Fig. S7A), suggesting that low AMPKα1 expression predicts a favorable response to oxaliplatin-based chemotherapy. Patients with response to oxaliplatin-based chemotherapy tends to showed lowered AMPKα1 expression than those resistant to oxaliplatin (Fig. S7B). Strikingly, the progression-free survival of patients with low AMPKα1 protein expression was dramatically longer than that of patients with high AMPKα1 expression (*P* < 0.05; Fig. S7C). We then generated three patient-derived xenografts to evaluate therapeutic potential of AMPKα1 silence. As shown in Figs. 6f and S8, combination of HA-quaternary/AMPKα1-shRNA3 and oxaliplatin significantly suppressed tumor growth compared with oxaliplatin alone in the mice.

On the basis of the above observations, we concluded that downregulating the expression of AMPKα1 could significantly inhibit tumor growth in subcutaneous cell-line-derived xenograft model and patient-derived xenograft model, which highly suggests the potential of AMPKα1 gene silencing as a therapeutic target for CRC treatment.

## Discussion

Maintaining metabolic homeostasis under conditions of energy stress is essential for cancer cell survival and induces cancer recurrence and metastasis following radical treatments. Our study aimed to address this important clinical issue and found that AMPKα1 may be a predictor for the prognosis of CRC patients. Mechanistic studies show that AMPKα1 is critical for CRC cell survival, and deletion of AMPKα1 renders cancer cells susceptible to physiological damage by ROS via alterations in GSR phosphorylation and a subsequent decrease in reduced glutathione. In some genetic analyses, AMPK has been shown to behave as a tumor suppressor and a number of drugs that activate AMPK also suppress cell growth [21, 22]. However, several studies have indicated that physiological AMPK activation is pro-tumorigenic [18]. Previous studies have not been performed on samples from patients that represent the in vivo physiological microenvironment. Our results are consistent with the findings from studies on myeloid leukemia in which AMPK was shown to protect leukemia-initiating cells from metabolic stress in bone marrow [19]. CRC shows an adaptive response to hypoxia via an upregulation of anaerobic glycolysis and angiogenesis [23]. Our results demonstrate that AMPKα1 can aide in the survival of CRC cells under physiological metabolic stress, which may lead to disease recurrence as well as a poor prognosis.

Our results further support the notion that AMPKα1 and AMPKα2 may have different functions. These two isoforms of AMPKα have shown specific effects in normal tissues and may also perform different functions in cancer cells [17, 24, 25]. In humans, AMPKα1 is ubiquitously distributed while AMPKα2 is abundant in skeletal and cardiac muscle [26]. We observed uniformly higher expression levels of AMPKα1 in patients’ samples and CRC cell lines than in nontumorous samples and normal cell lines; while the expression levels of AMPKα2 were quite low in patients’ samples. Higher expression of AMPKα1 in tumors than in normal tissue was also observed in other cancer types such as pancreatic and cervical cancer [27, 28]. Silencing of AMPKα1 with RNA interference inhibited the growth of pancreatic cancer cells in vitro and in vivo [28]. In chondrosarcoma cells and pancreatic cancer cells, the inhibition of both AMPKα1 and AMPKα2 reduced cell migration and tumor growth [26, 29]. The role of AMPKα1 may vary due to the different cell types in which the cancer was initiated. Our results suggest that different roles might be played by AMPKα1 and AMPKα2 in CRC. Loss of AMPKα1 expression has also been associated with poor survival in melanoma patients, which suggests AMPKα1 may perform different roles in different tumor types [30].

Cancer cells frequently exhibit high levels of oxidative stress and an upregulation in their antioxidant capacity [31]. On one hand, the elevation of ROS in cancer promotes cell proliferation, cell survival, and tumor development [32]. On the other hand, when the increase in ROS reaches a certain level, it may overwhelm the antioxidant capacity of the cells and trigger cell death [31]. Glutathione is the most abundant antioxidant in cells and acts to maintain redox balance [33]. Compounds which target the glutathione antioxidant system and cause severe ROS accumulation have been shown to preferentially kill cancer cells and prolong animal survival [34–36]. Current phosphorylation profiling has identified several phosphorylation sites in GSR [37, 38]. However, the function and regulatory mechanism of these sites remain unknown. Our study, for the first time, identified AMPKα1 as an upstream regulator of GSR possibly through phosphorylation at residue Thr507 and thus increase its activity.

To evaluate the therapeutic effect on AMPKα1, we specifically developed a modified PPMS/shRNA polyplexes for efficient systemic gene delivery to manipulate the gene expression levels of AMPKα1 in CRC models. Our results clearly demonstrated that the HA-quaternary polyplexes could effectively deliver AMPKα1 shRNA to CRC cells in vivo and that downregulation of the expression of AMPKα1 could significantly inhibit tumor growth in subcutaneous CRC models. We also found AMPKα1 inhibition is synergistic with oxaliplatin both in cell-line-derived xenograft model and patient-derived xenograft model. This study proved that AMPKα1 could be potentially used as a therapeutic target for CRC treatment. But it still need further study to investigate the optimal way of AMPKα1 inhibition in humans.

In conclusion, our study demonstrates that the upregulation of AMPKα1 in CRC promotes cancer cell survival under conditions of energy stress and leads to a poor prognosis through maintaining cellular glutathione by regulating glutathione reductase phosphorylation. The strategy of AMPKα1 inhibition may be applied to treat CRC patients.

## Materials and methods

### Patients, samples, and immunohistochemical analysis

Fresh tissue samples from 140 patients with stage II - III CRC from Sun Yat-sen University Cancer Center were stored in liquid nitrogen immediately after surgery until used for analysis. All samples were authenticated by hematoxylin and eosin (H&E) staining and those with tumor cells more than 40% were subjected to protein extraction using radioimmunoprecipitation assay (RIPA) buffer (Cell Signaling Technology, MA, USA). A functional proteomic analysis of these samples was conducted via RPPA analysis. Meanwhile, formalin-fixed paraffin-embedded tissues of a cohort containing 346 patients were used for the immunohistochemical analysis of AMPKα1 according to previously reported protocols [39]. The tumor tissues of 76 patients with advanced CRC treated with FOLFOX or XELOX regimens were collected from Sun Yat-sen University Cancer Center. The AJCC criteria were used to classify the clinical and clinic-pathological stages and patient consent and approval was obtained from the Institutional Research Ethics Committee.

To quantify AMPKα1 or pGSR(T507) protein expression, both the intensity and extent of immunoreactivity were evaluated and scored. In the present study, IHC intensity was scored as follows: 0, negative staining; 1, weak staining; 2, moderate staining; 3, strong staining. The scores of the extent of immunoreactivity ranged from 0 to 3 and were according to the percentage of cells that had positive staining in each microscopic field of view (0, <25%; 1, 25–50%; 2, 50–75%; 3, 75–100%). A final score ranging from 0 to 9 was achieved by multiplying the scores for intensity and extent. AMPKα1 or pGSR(T507) expression levels were considered high when the final scores were ≥4 and low when the final scores were <4.

### Cell lines and cell culture

Human CRC cell lines SW480, HCT116, SW1116, SW620, HCT8, HT-29, DLD-1, RKO, Ls174T, and LoVo and the colon epithelial cell line CCD112, CCD841 were purchased from the American Type Culture Collection (Manassas, VA, USA) in 2009 and cultured according to the instructions with 10% FBS (Thermo Fisher Scientific, CA, USA) supplementation. All cells were cultured at 37 °C with 5% CO_2_. All the above cells were authenticated by short tandem repeat DNA fingerprinting and tested for mycoplasma before using. Glucose-free media were obtained from Life Technology (CA, USA).

### Reagents and antibodies

Purified D-glucose and DCF-DA were obtained from Life Technologies. Oxaliplatin and NAC were purchased from Selleck Chemicals (TX, USA). Antibodies used for immunoblotting included those for AMPKα1 (Millipore, MA, USA, MABS818), pAMPK(Thr172) (Cell Signaling Technology, MA, USA, #2535), AMPKα2 (CST, #2725), GSR (Abcam, Cambridge, MA, USA, ab16801), vinculin (Abcam, ab129002), GAPDH (ab128915), and β-actin (CST, #4970).

### Real-time PCR

Total RNAs were obtained with TRIzol reagent (Life Technologies, CA, USA) and reverse transcribed to cDNA using a PrimeScript RT Master Mix kit (Takara, NHK, Japan). mRNA expression levels were detected using real-time PCR according to a previous report [39]. Synthesized primers from Life Technologies were as follows:

AMPKα1-Forward: GGCACGCCATACCCTTGAT

AMPKα1-Reverse: TCTTCCTTCGTACACGCAAATAA

AMPKα2-Forward: GTGAAGATCGGACACTACGTG

AMPKα2-Reverse: CTGCCACTTTATGGCCTGTTA

GAPDH-Forward: ATCACCATCTTCCAGGAGCGA

GAPDH-Reverse: CCTTCTCCATGGTGGTGAAGAC

### Western blot

Proteins were extracted with RIPA buffer and quantized using a BCA assay (Thermo Fisher Scientific, CA, USA). A total of 40 μg were loaded and analyzed by western blotting as previously described [39, 40].

### Cell apoptosis, cell death, and proliferation assays

Cell apoptosis induced by culturing with glucose-free medium was determined by annexin V/PI staining (4A Biotech Co, Beijing, China) followed by flow cytometry, according to the manufacturer’s instructions. A trypan blue exclusion assay was conducted to detect cell death after exposure to glucose-free medium at the indicated time points. CCK-8 and colonic assay were used to detect cell viability and proliferation as described previously [41, 42].

### Plasmid

The opening read frame of GSR (NM_000637) was cloned to the plasmids of pCMV6-Flag-empty (Genecopoeia^TM^, MA, USA) and denominated as (GSR-WT). The targeted point mutation of GSR including T507V, T507D, and T507E was generated with the MutanBEST kit (Takara, NHK, Japan) according to the manufacturer’s instructions.

### Immunoprecipitation

Cells were rinsed twice with ice-cold PBS and lysed in ice-cold lysis buffer (50 mM Tris [pH 8.0], 150 mM NaCl, 5 mM EDTA, 0.1% NP40) and 1% of protease inhibitors (Selleck, TX, USA). The cell lysates were isolated by centrifugation at 13,000 rpm for 15 min. For immunoprecipitations, the Flag-beads (Sigma-Aldrich, MO, USA) were co-incubated with the soluble supernatant in 4 °C overnight. Immunoprecipitates were washed three times with lysis buffer. Cell extracts or immunoprecipitated proteins were denatured by the addition of loading buffer followed by boiling for 5 min, resolved by 10% SDS-PAGE, and then transferred to nitrocellulose-ECL membranes (Millipore, Darmstadt, German) and incubated with antibodies against phospho-(Ser/Thr) (Abcam, MA, USA). The immune complex was detected by chemiluminescence (Thermo Fisher Scientific, CA, USA).

### Enzyme assays

The enzymatic activities of GSR was measured with the Glutathione Reductase Activity Colorimetric Assay Kit (Biovision, CA, USA) and conducted according to the manufacturer’s instructions. Briefly, the cells transfected with the indicated vectors were homogenized on ice in four volumes of cold assay buffer and the supernatants were collected for assay after centrifugation at 10,000 × *g* for 15 min. The samples were mixed with catalase, incubated with reaction buffer after depletion of endogenic glutathione. The colorimetric intensity of samples was detected on a Synergy™ Multi-Mode Microplate Reader (Biotek, VT, USA) at a wavelength of 405 nm. The GSR activity in the samples was calculated according to the standard curve.

### In vivo tumor growth

Female athymic BALB/c nude mice (4–5 weeks of age, 15–18 g) were purchased from Guangdong Province Laboratory Animal Center. All animal studies were performed in accordance with institutional and international animal regulations. Randomization was conducted and mice were treated by an unblinded manner. Animal protocols were approved by the Institutional Animal Care and Use Committee of Sun Yat-sen University Cancer Center. To evaluate whether knockdown of AMPKα1 could inhibit tumor growth, 1 × 10^6^ RKO or HCT116 cells expressing control-shRNA (sh-NC), AMPKα1-shRNA2 (sh-2), or AMPKα1-shRNA3 (sh-3) were subcutaneously injected into the right flank of the mice (*n* = 6). Tumor volumes were measured on day 7 and every other day from day 12. The tumor volume was calculated with the equation: **V**(mm^3^) = **a** × **b**^2^/2, where **a** is the longest diameter and **b** is the shortest diameter. Twenty days later, mice were sacrificed and tumors were dissected out and prepared for paraffin-embedded sectioning.

### NADPH, GSH, and ATP assays

The intracellular levels of GSH were measured with the GSH-Glo™ kit (Promega, WI, USA). Briefly, 8000 cells were incubated in a 96-well plate. The next day, the medium was removed, rinsed twice with PBS and glucose-free medium was added. A total of 100 μL of 1 × GSH-Glo™ reagent were then added into the 96-well plate followed by the removal of the medium 12 h later. With slight shaking, the well was incubated for 30 min at room temperature. Equal volumes of reconstituted luciferin-detection reagent were then added into each well and samples were incubated for an additional 15 min at room temperature. Luminescence detection in a micro-well reader was then conducted. The intracellular levels of NADPH, total NADP (NADPH + NADP^+^), and ATP were measured with the NADP/NADPH-Glo^TM^ kit and the CellTiter-Glo^®^ kit (Promega, WI, USA), respectively, according to the manufacturer’s instructions.

### SiRNA transfection and lentivirus transduction

Specific siRNA targeting AMPKα1 (Ribobio, Guangzhou, China) were transfected into the indicated cells using Lipofectamine 2000 (Invitrogen, CA, USA) according to the manufacturer’s instructions. Target sequences used were as follows: AMPKα1 siRNA1, GAGGAGAGCTATTTGATTA; AMPKα1 siRNA2, GCAGAAGTATGTAGAGCAA. The cells were harvested 48 h post transfection for ROS detection and apoptosis analysis or for western blotting to verify and quantify the knockdown efficiency. Stable AMPKα1 and GSR knockdown cells were generated as previously described [43] and the target sequences were as follows:

AMPKα1-shRNA1, CGGCAAAGTGAAGGTTGGCAAACAT;

AMPKα1-shRNA2, GCTGCACCAGAAGTAATTTCA;

AMPKα1-shRNA3, GACCTCACTTGACTCTTCTCCTGTT;

GSR-shRNA, CCAAGTTGTGAGGGTAAAT.

### Assay for reactive oxygen species

The intracellular levels of ROS were measured with 5-(and-6)-Carboxy-2’,7’-Dichlorofluorescein Diacetate (DCF-DA). Cells (1 × 10^5^) were plated in a 12-well plate and were transfected with specific siRNA, cultured with 5 mM glucose or glucose-free medium and were treated with DCF-DA for 30 min. The cells were then washed with PBS and collected as single-cell suspensions. Cell fluorescence was detected by flow cytometry.

### Statistics

All data are presented as the mean ± SD. The immunohistochemical analysis about CRC tissue samples was performed by a blinded manner. To compare the significant differences of more than two groups, one-way ANOVA and Newman–Keuls multiple comparison tests were used. Other statistical analyses were performed using the unpaired Student’s *t*-test (GraphPad Prism). For survival evaluation, the Kaplan–Meier method was used to investigate the correlation between variables and overall survival, while a log-rank test was employed to compare survival curves. We also used a Cox regression model to perform the univariate and multivariate survival analyses. All of the above methods for survival analysis were performed with the survival package of R software and the other statistical tests were performed with R software version 3.1.0. The variance between the groups that are statistically compared is similar. Statistical significance was set at 0.05.

## Supplementary information

Supplementary files
