# Supplementary material for: AMPKα1 confers survival advantage of colorectal cancer cells under metabolic stress by promoting redox balance through the regulation of glutathione reductase phosphorylation
Source: Oncogene. 2019 Sep 17;39(3):637–50. doi: 10.1038/s41388-019-1004-2 (PMC6962094; doi:10.1038/s41388-019-1004-2)
Supplement: Supplementary file 1 — Supplementary files [file 41388_2019_1004_MOESM1_ESM.pdf]

Supplementary method

Reverse phase protein arrays

RPPA data normalization

Protein digestion and tandem mass spectrometry

Liquid chromatography/mass spectrometry (LC/MS) and metabolomic analysis

In vitro gene transfection

In vivo antitumor efficacy

Supplementary Figures and Tables

Figure S1. Expression of AMPKα1 in CRC cell lines.

Figure S2. AMPKα1 knockdown reduces SW1116 and DLD1 cells survival in the absence of glucose.

Figure S3. Overexpression of GSR mutant in AMPKα1 and GSR knockdown cells.

Figure S4. Characterization of the polyplexes.

Figure S5. Therapeutic efficiency of HA-Quaternary mediated AMPKα1 knockdown and oxaliplatin *in vivo*.

Figure S6. Therapeutic efficiency of Compound C and oxaliplatin *in vivo*.

Figure S7. High AMPKα1 expression predict a worse response and PFS in advanced CRC patients.

Figure S8. Knockdown of AMPKα1 suppressed tumor growth in PDX model.

Table S1. Baseline characteristics of patients tested in reverse phase protein array (RPPA) assay.

Table S2. Univariable associations of 13 proteins with overall survival from RPPA

data.

Table S3. Multivariable Cox regression analysis of overall survival in patients with stage II or III colorectal cancer.

Table S4. The IC50 of CRC cell lines to oxaliplatin.

Supplementary method

Reverse phase protein arrays

Fresh tissues were verified by professional pathologists and those with tumor percentages more than 40% underwent RPPA analysis. Briefly, tissues were set in a weighing dish at room temperature, cut into small pieces and placed in 2-ml tubes with ceramic beads and cold lysis buffer appropriate for homogenization in a Precellys homogenizer. After homogenization, tubes were centrifuged at 4°C for 15 minutes at 14,000 rpm. The supernatant was collected and the protein concentration was determined by BCA assay and adjusted to 1-1.5 μg/μl. The cell lysates were mixed with a sample buffer without bromophenol blue and boiled for 5 minutes. Samples were submitted for RPPA processing according to standard procedures.

RPPA data normalization

Using an RPPA assay, we examined 141 cancer-related proteins and phosphoproteins in a total of 140 tumor-normal paired samples. The raw data matrix was normalized by protein loading, which has been described previously 40. The normalization steps are as follows: 1) Convert the raw data from log 2 values to linear values. 2) Determine the median for each antibody across the sample set. 3) Divide each raw linear value by the median within each antibody to obtain the median-centered ratio. 4) Calculate the median of the median-centered ratios (from Step 3) for each sample across the entire panel of antibodies. This median functions as the correction factor (CF.1) for protein loading adjustments, and each sample has its own correction factor. Samples with correction factors above 2.5 or below 0.25 are considered outliers. 5) Divide each

median-centered ratio for the linear values (from Step 3) by the correction factor (CF.1 from Step 4) to obtain the normalized linear value.

After data normalization, 4 samples from 2 patients were identified as outliers during the RPPA data quality control step (correction factor was above 2.5 or below 0.25) and removed and 36 samples from 18 patients were removed due to incomplete survival data. Therefore, a matrix with 141 rows and 240 columns was retained for further analysis. To perform a survival analysis at the individual level, the relative protein expression value of each tumor sample was obtained by subtracting the value in the corresponding normal sample to reduce the noise from stromal cells in the tumor. Eventually, a new data matrix that consisted of 141 variables and 120 cases was generated as the input file used for comparisons and survival analysis.

Protein digestion and tandem mass spectrometry

Immuoprecipitates from HCT116 shNC and AMPKα1-shRNA3 cells cultured under glucose-deprived conditions for 4 hours were separated by SDS-PAGE electrophoresis. The bands with molecular weight at 58kDa were cut off and subjected to trypsin digestion. Briefly, the bands were destained with destaining solution (100mM NH4HCO3 in 30%v/v ACN) and the destaining step was repeated until the gel was colorless. After washing with 100mM NH4HCO3, the gel pieces were rehydrated in 100mM NH4HCO3 with 10ng sequencing grade-modified trypsin (Promega, Madison, WI, USA) at 37°C overnight. After digestion, the protein peptides were collected, and the gels were washed with 0.1% TFA in 60% ACN to collect the remaining peptides. Then the samples were analyzed using Easy-nLC nanoflow HPLC system connected to

Orbitrap Elite Mass spectrometer (Thermo Fisher Scientific, CA, USA). A total of 1μg of each sample was loaded onto Thermo Scientific EASY column (two columns) using an autosampler at a flow rate of150nL/min. The sequential separation of peptides on Thermo Scientific EASY trap column (100μm × 2cm, 5μm, 100Å, C18) and analytical column (75μm×25 cm, 5μm, 100 Å, C18) was accomplished using a segmented 1h gradient from Solvent A (0.1% formic acid in water) to 50% Solvent B (0.1% formic acid in 100% ACN) for 50 min, followed by 50-100% Solvent B for 4 min and then 100% Solvent B for 6 min. The column was re-equilibrated to its initial highly aqueous solvent composition before each analysis. The mass spectrometer was operated in positive ion mode, and MS spectra were acquired over a range of 300-2000 m/z. The resolving powers of the MS scan and MS/MS scan at 200 m/z for the Orbitrap Elite were set as 60,000 and 15,000, respectively. The top ten most intense signals in the acquired MS spectra were selected for further MS/MS analysis. The isolation window was 1m/z, and ions were fragmented through higher energy collisional dissociation with normalized collision energies of 35eV. The maximum ion injection times were set at 50ms for the survey scan and 150ms for the MS/MS scans, and the automatic gain control target values for full scan modes was set to 1.0×10−6 and for MS/MS was 5 × 104. The dynamic exclusion duration was 30s. The raw files were analyzed using the Proteome Discoverer 1.4 software (Thermo Fisher Scientific, CA, USA). Search for the fragmentation spectra was performed using the MASCOT search engine embedded in Proteome Discoverer against the self-organization database (Uniprot Homo sapiens 154578). The following search parameters were used: monoisotopic mass, trypsin as

the cleavage enzyme, two missed cleavages, carbamidomethylation of cysteine as fixed modifications, and peptide charges of 2+, 3+, and 4+, and the oxidation of methionine was specified as variable modifications. The mass tolerance was set to 20 ppm for precursor ions and to 0.05 Da for the fragment ions. The results were filtered based on a false discovery rate (FDR) of no more than 1%.

Liquid chromatography/mass spectrometry (LC/MS) and metabolomic analysis

Cell sample collection and preparation for hydrophilic interaction chromatography (HILIC) LC/MS analysis were performed according to our previously reported methods 42. Five microliters of the prepared sample was injected and analyzed using an Ultimate 3000 UHPLC system (Dionex Corporation, CA, USA) interfaced with a Q Exactive mass spectrometer (Thermo Fisher Scientific, CA, USA). The column temperature and all samples were maintained at 40°C and 4°C, respectively, prior to their mass spectrometry-based metabolomics analysis. Chromatographic separations were performed on a Waters Atlantis® HILIC Silica 3-µm column (2.1 × 100 mm, Waters Corp, Milford, MA). The mobile phase consisted of solvent A (50% acetonitrile+10 mM NH4FA+0.1% formic acid) and solvent B (95% acetonitrile +10 mM NH4FA+0.1% formic acid). The gradient program was as follows: 0 min 100% B, 1 min 100% B, 20 min 0% B, 24.9 min 0% B, 25 min 100% B, 30 min 100% B, 30 min stop. The flow rate was 0.3 mL/min and the injection volume was 5 µL. The samples were independently examined in both positive and negative ionization modes using a dual electrospray ionization (ESI) source. The spray voltages were set to 3.5/2.8 kV (+/-). Capillary and auxiliary gas heater temperatures were set at 325°C and 350°C,

respectively. Nitrogen was used as both the sheath gas (flow rate 40 arb) and auxiliary gas (flow rate 10 arb).

The high-resolution accurate-mass (HRAM) data acquired from Q Exactive mass spectrometer were aligned and extracted by using SIEVE 2.2 (Thermo Fisher Scientific, MA, USA). The multivariate data matrix was further exported into SIMCA-P+13 software (Umetrics, NJ, USA). Unsupervised principal components analysis (PCA) and supervised orthogonal partial least squares discriminant analysis (OPLS-DA) models were constructed to compare the cell metabolomes. A list of ions showing considerable group-discriminating power was generated from the loading S-plot and screened with VIP>1. Ions that were significantly altered in different groups were further identified by comparing the retention times and fragmentation patterns with a standard database from METLIN (http://metlin.scripps.edu).

***In vitro* gene transfection**

The gene silencing efficiency of the HA-Quanternary loaded AMPKα1-shRNA3 was assessed by quantitative real-time PCR and western blot. HCT116 cells were dispensed on a 6-well plate at a density of 3×10^5 cells per well and incubated for 24h. Binary and HA-Quanternary polyplexes containing 3.5 μg of control-shRNA/AMPKα1-shRNA3 were incubated with cells for 48 h. Total mRNA was isolated and 1μg cDNA was used to qRT-PCR. Protein in transfected cells was isolated by 100 μL of RIPA buffer. The cell lysis was performed on ice for 30 min and the supernatant of lyses were collected after centrifugation for 15 min at 12,000 rpm. The concentration of protein was determined using the BCA protein assay method at 562 nm. LipofectamineTM

2000 (Thermo Fisher Scientific, CA, USA) was used as a positive control.

***In vivo* antitumor efficacy**

Female athymic BALB/c nude mice (4–5 weeks of age, 15-18g) were purchased from Guangdong Province Laboratory Animal Center. Randomization was conducted and mice were treated by an unblinded manner. To establish subcutaneous tumor model, mice were injected with 1×10^6 HCT116 or RKO cells/mouse (n = 5-6). When the tumor volume had reached approximately 50-100 mm^3, tumor bearing mice were randomly assigned to four groups: vehicle control, HA-Quaternary/AMPKα1-shRNA3 or Compound C, oxaliplatin, combination. For PPMS polyplexes, shRNA dosages were set as 20 μg per mouse. For Compound C treatment, the dosage was 15 mg/kg.

Gene therapy treatment in PDX (Patient-derived xenograft) was conducted 5 times through tail vein injection every 3 days; the day of the first administration was recorded as day 0. Tumor volumes and body weights were recorded every 3 days. The tumor volume was calculated with the equation: $V(mm^3) = a \times b^2/2$, where a is the longest diameter and b is the shortest diameter. After 18 days, mice were sacrificed and tumors were dissected out and prepared for paraffin-embedded sectioning. The protein and mRNA were extracted from the tumor tissue for AMPKα1 protein analysis.

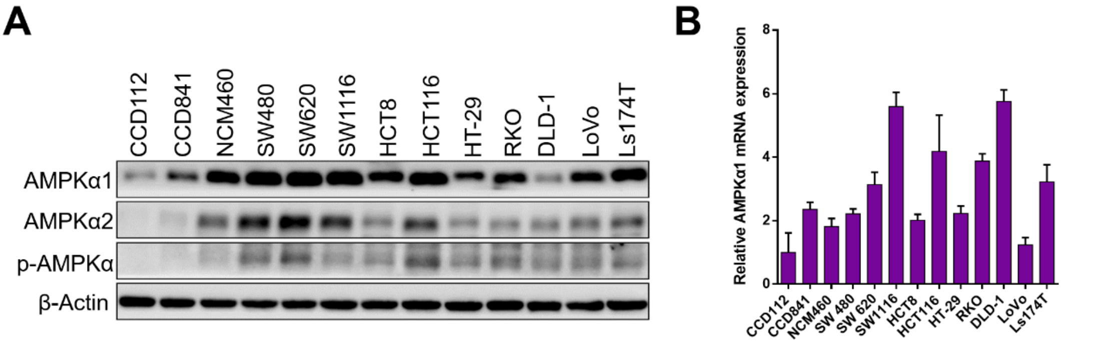

Figure S1. Expression of AMPKα1 in CRC cell lines. Related to Figure 1. (A) Expressed AMPKα1, AMPKα2 and p-AMPKα were immunoblotted in a panel of 11 CRC cell lines and immortalized colon epithelial cell lines (CCD112, CCD841 and NCM460). (B) mRNA expression of AMPKα1 was examined with qRT-PCR and indicated an up-regulation in CRC cell lines.

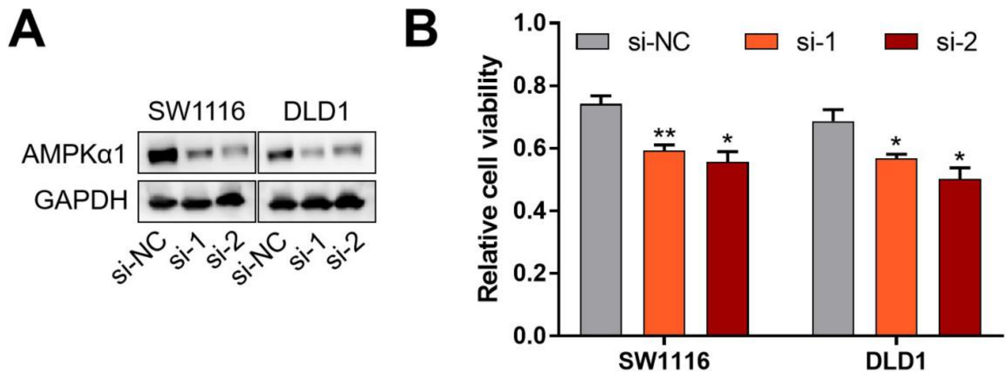

Figure S2. AMPKα1 knockdown causes SW1116 and DLD1 cell death in the absence of glucose. Related to Figure 2. (A) Immunoblotting of AMPKα1 transfected with siRNA in SW1116 and DLD1 cells. (B) Viability of SW1116 and DLD1 cells was detected by CCK-8 assay in the absence of glucose for 48 h.

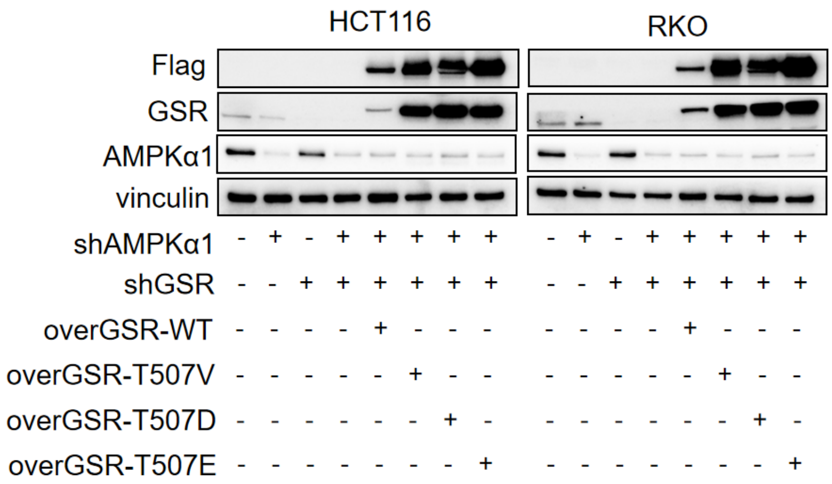

Figure S3. Overexpression of GSR mutant in AMPKα1 and GSR knockdown cells. Related to Figure 5. Wesstern blot showed the knockdown of AMPKα1 and GSR and the overexpression of mutant GSR in AMPKα1 and GSR double knockdown cells.

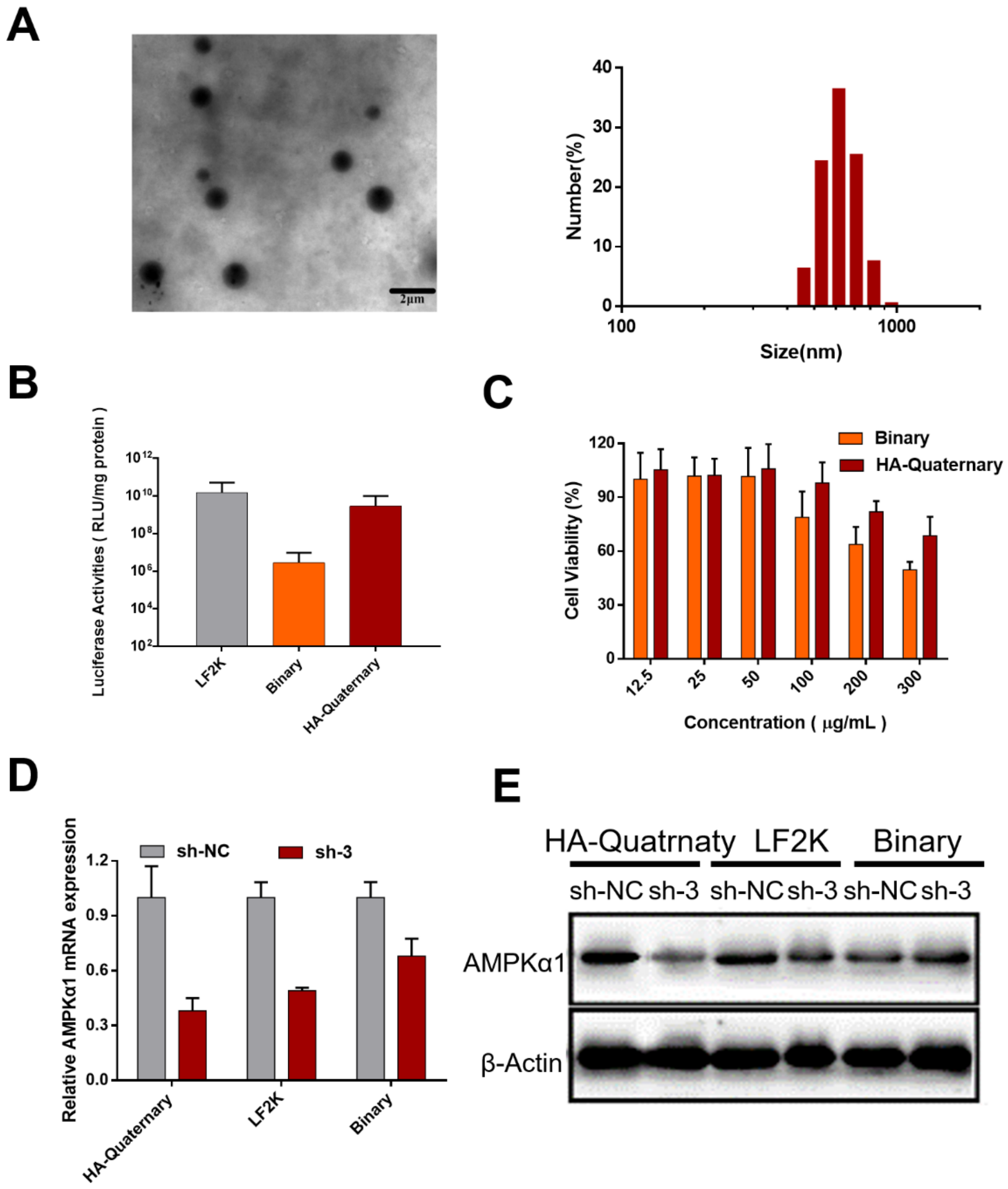

Figure S4. Characterization of the polyplexes. Related to Figure 6. We have developed a series of biodegradable poly(amine-*co*-esters) through enzymatic copolymerization, many of these cationic poly(amine-*co*-esters), particularly poly (*ω*-pentadecalactone-*co*-*N*-methyldiethyleneamine-*co*-sebacate) (PPMS) copolymers exhibited good performance as gene delivery vector. To further enhance the targeting capability and gene transfection efficiency of the polyplexes, the nuclear targeting peptides NLS and the surface coating of HA-PEG were incorporated into this

PPMS/shRNA binary polyplexes. Due to the modification of the HA, The quaternary polyplex had a dramatically decreased zeta potential of 2.2 mV compared with binary polyplex which possessed a zeta potential of 57 mV. (A) The TEM image and zeta-size measurement indicated that the HA-quaternary polyplex were spherical in shape and possessed a hydrodynamic size distribution between 500 nm to 800nm. (B) At optimized polyplex transfection condition, the gene transfection efficiency of the HA-quaternary polyplex was almost a thousand folds higher than binary polyplex. It is likely attributable to the increased cellular uptake by HA modification and enhanced intracellular transport of DNA into the nucleus by NLS incorporation. (C) The HA-PEG layer on HA-quaternary polyplexes could not only enhance the nanoparticle stability in physiological environment but also decrease the cytotoxicity at the higher polyplex concentration (> 100μg/mL). (D and E) *In vitro* AMPKα1 gene knockdown efficiency was evaluated on HCT116 cells using HA-quaternary polyplex loaded with AMPKα1-shRNA. After 48 h transfection, the HCT116 cells were harvested and the expression of AMPKα1 mRNA and protein were measured by RT-PCR and western blot. The results showed that the HA-quaternary/AMPKα1-shRNA3 resulted in a significant downregulation of AMPKα1 on both mRNA level (D) and protein level (E) compared with binary polyplexes group.

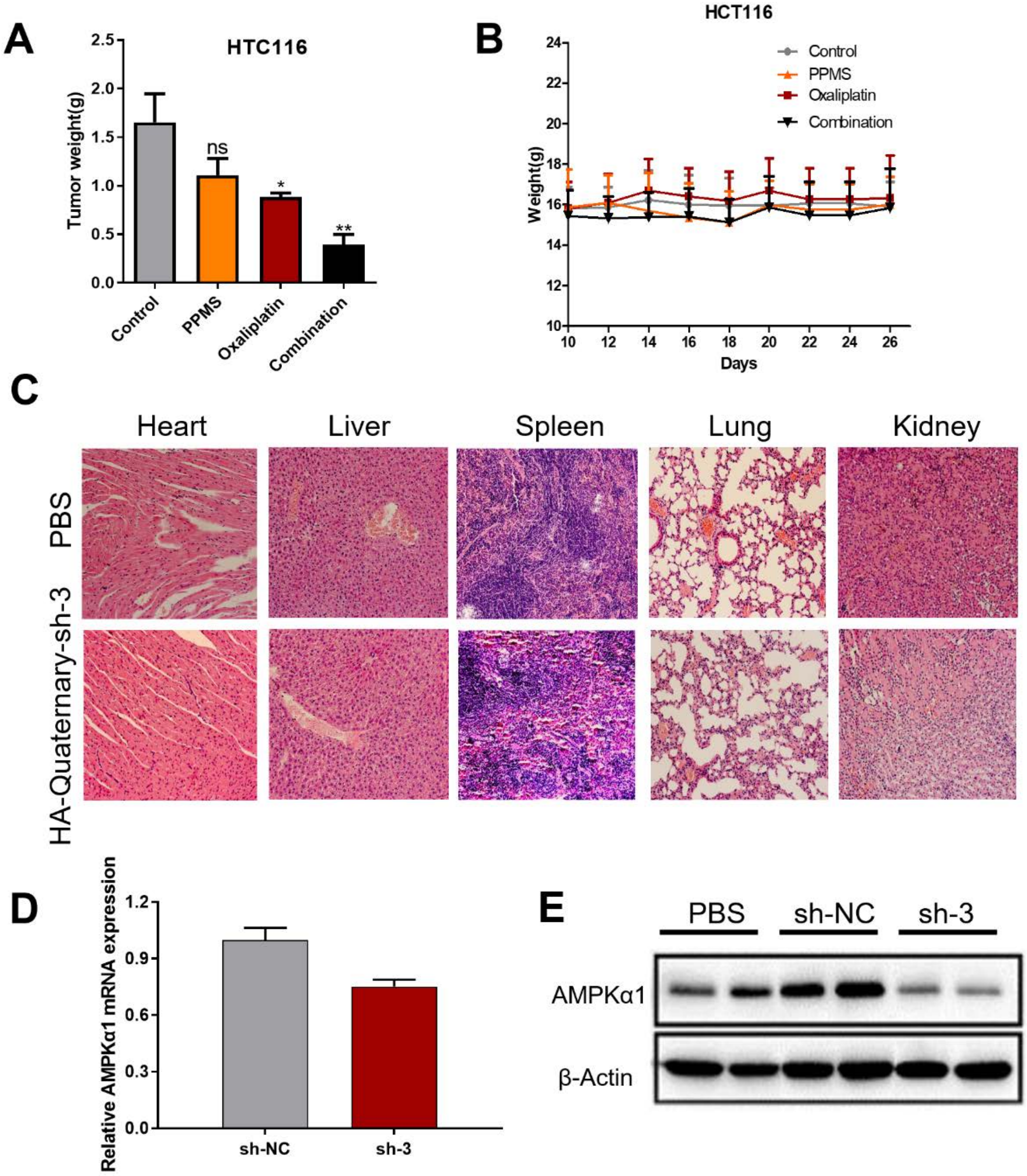

Figure S5. Therapeutic efficiency of HA-Quaternary mediated AMPKα1 knockdown and oxaliplatin *in vivo*. Related to Figure 6. Dissected tumor weight (A) and mouse weight (B) of HCT 116 cells based xenografts treated with PPMS, oxaliplatin or both agents. (C) Representative organ histology for PBS, HA-Quaternary/sh-3 injected mice by H&E staining. *In vivo* gene transfection efficiency shown by AMPKα1 mRNA (D) and protein (E) level in xenografts treated with HA-quaternary/sh-NC or HA-quaternary/sh-3.

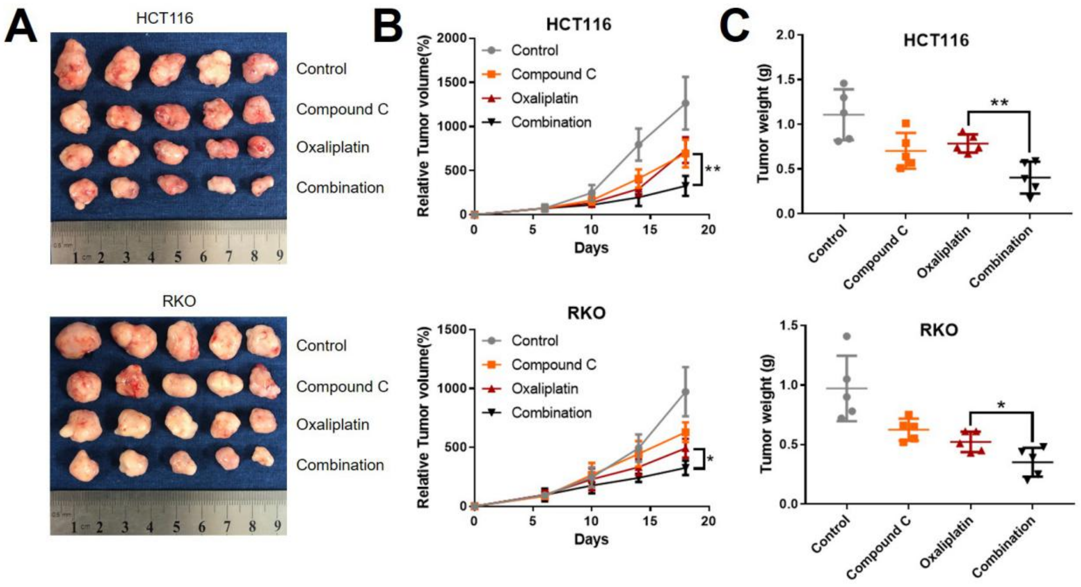

Figure S6. Therapeutic efficiency of Compound C and oxaliplatin *in vivo*. Related to Figure 6. HCT116 and RKO cells were subcutaneously injected into nude mice and randomly separated the mice into 4 groups (n = 5 for each group). When the tumor volume reached approximately 100 mm^3, the groups were treated with vehicle, oxaliplatin, compound C or combination therapy every 3 days. After 4 treatments, the mice were euthanized, and their tumors were excised. (A) The images of dissected tumor xenografts after treatments as indicated. (B) Tumor volumes after treatment by Compound C, oxaliplatin, or combination therapy until day 18. (C) Tumor weights of the indicated groups in (B).

A

FOLFOX or XELOX		AMPKα1 expression			*P* value
		High	Low	Total	
Response	CR+PR+SD	26	27	53	*P* = 0.023
	PD	18	5	23	
	Total	44	32	76	

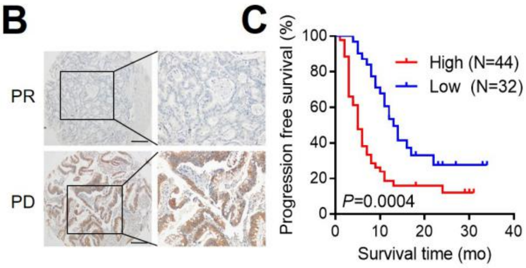

Figure S7. High AMPKα1 expression predict a worse response and PFS in advanced CRC patients. Related to Figure 6. A cohort of 76 CRC patients were received 1st line oxaliplatin-based chemotherapy FOLFOX or XELOX who were enrolled from our institution. (A) Percentage of specimens with low or high AMPKα1 expression, relative to the response to oxaliplatin-based chemotherapy analyzed again by Pearson Chi square test. (B) Two representative cases of AMPKα1 expression are shown. Scale bar: 200μm. (C) The progression-free survival curves of patients with low and high AMPKα1 expression are generated using the Kaplan–Meier method and the Log-rank test. PD, progressive disease; CR, complete response; PR, partial response; and SD, stable disease.

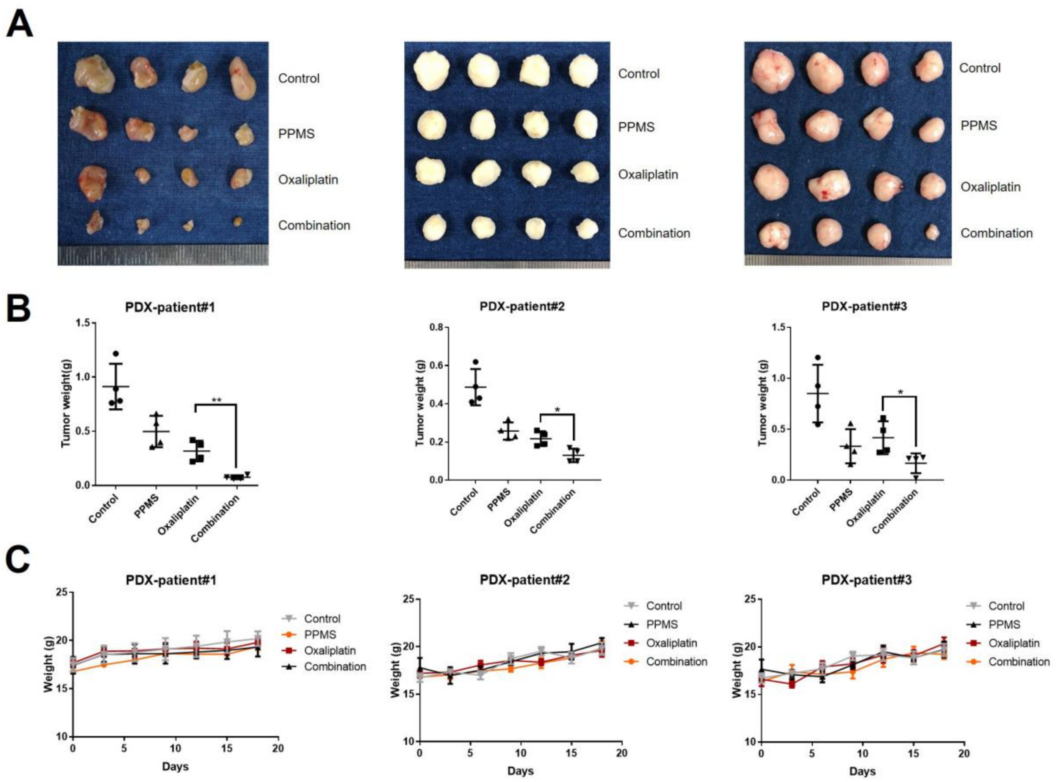

Figure S8. Knockdown of AMPKα1 suppressed tumor growth in PDX model. Related to Figure 6. (A) The images of dissected tumor xenografts after the indicated treatments. (B and C) Dissected tumor weight (C) and mouse weight (D) of patient derived xenografts treated with PPMS, oxaliplatin or both agents.

Table S1 Baseline characteristics of patients tested in reverse phase protein array (RPPA) assay.

		Number of patients (n=120)	%
**Gender**			
Male		73	61%
Female		47	39%
**Age**			
65 years or younger		86	72%
Older than 65 years		34	28%
**Stage II (TNM, AJCC)**		57	48%
Chemotherapy	Yes	32	56%
	No	25	44%
pAMPK (T172) levels (upregulated and downregulated in tumor)	High	42	67%
	Low	15	33%
**Stage III (TNM, AJCC)**		63	52%
Chemotherapy	Yes	54	86%
	No	9	14%
pAMPK (T172) levels (upregulated and downregulated in tumor)	High	48	76%
	Low	15	24%

Table S2 Univariable associations of 13 proteins with overall survival from RPPA data.

We tested 141 proteins in total. Only 13 variables were significantly correlated with overall survival (Wald test P value < 0.05). According to the C-index of each protein, we found pAMPK(T172) shows the best discriminatory ability of random effects for overall survival status.

Proteins	Hazard Ratio(CI 95%)	P	C-index
**AMPK_pT172**	2.4986 (1.3785-4.5288)	0.0025	0.6521
**eIF4E**	8.0905 (1.7032-38.4311)	0.0085	0.6351
**STAT5-alpha**	3.0937 (1.0880-8.7965)	0.0342	0.6270
**MEK1**	174.7346 (1.3134-23246.7027)	0.0385	0.6109
**eEF2K**	21.6802 (1.2539-374.8425)	0.0344	0.6025
**B-Raf**	14.9567 (1.6897-132.3941)	0.0150	0.6019
**14-3-3_beta**	0.0000 (0.0000-0.1869)	0.0273	0.5968
**p27_pT198**	0.0000 (0.0000-0.1439)	0.0182	0.5938
**Claudin-7**	1.3062 (1.0198-1.6730)	0.0344	0.5700
**MYH11**	1.0092 (1.0011-1.0174)	0.0255	0.5546
**Rictor_pT1135**	0.0040 (0.0001-0.1434)	0.0025	0.5529
**IGFBP2**	2.9211 (1.2173-7.0096)	0.0164	0.5375
**YAP**	76.2868 (1.1863-4905.8016)	0.0413	0.4822

Table S3. Multivariable Cox regression analysis of overall survival in patients with stage II or III colorectal cancer.

**Characteristics**	**Coefficient**	**Hazard Ratio(CI 95%)**	**P value**
**Gender**			
(Male vs female)	0.063	1.065 (0.503-2.253)	0.87
**Age**			
(>65 vs <65)	0.705	2.024 (0.965-4.243)	0.062
**Chemotherapy**			
(Yes vs no)	0.032	1.033 (0.384-2.775)	0.949
**TNM Stage (AJCC)**			
III vs II	1.271	3.563 (1.423-8.920)	**0.007**
**AMPK_pT172**	0.860	2.364 (1.284-4.351)	**0.006**

Table S4. The IC50 of CRC cell lines to oxaliplatin.

We detected the IC50 of oxaliplatin in 10 CRC cell lines treated with oxaliplatin in three independent experiments.

IC50 (μM)		
**Cell line**	**mean**	**SD**
RKO	8.56	1.87
HCT116	7.65	2.09
LS174T	33.44	11.76
SW620	11.33	3.43
DLD1	56.78	11.90
SW1116	62.58	2.79
HT29	17.19	4.54
Lovo	18.36	10.18
SW480	19.61	2.72
HCT8	39.25	3.28
